# A peptidoglycan *N*-deacetylase specific for anhydroMurNAc chain termini in *Agrobacterium tumefaciens*

**DOI:** 10.1016/j.jbc.2023.105611

**Published:** 2023-12-28

**Authors:** Michael C. Gilmore, Akhilesh K. Yadav, Akbar Espaillat, Andrea A. Gust, Michelle A. Williams, Pamela J.B. Brown, Felipe Cava

**Affiliations:** 1 Department of Molecular Biology and Laboratory for Molecular Infection Medicine Sweden, Umeå Centre for Microbial Research, SciLifeLab, Umeå University, Umeå, Sweden; 2 Academy of Scientific and Innovative Research (AcSIR), Ghaziabad, Uttar Pradesh, India; 3 Regulatory Toxicology Group, CSIR-Indian Institute of Toxicology Research (CSIR-IITR), Lucknow, Uttar Pradesh, India; 4 Department of Plant Biochemistry, Center of Plant Molecular Biology (ZMBP), Eberhard-Karls-University of Tübingen, Tübingen, Germany; 5 Division of Biological Sciences, University of Missouri-Columbia, Columbia, Missouri, USA

**Keywords:** peptidoglycan, deacetylase, anhydromuropeptide, lytic transglycosylase, *Agrobacterium tumefaciens*

## Abstract

During growth, bacteria remodel and recycle their peptidoglycan (PG). A key family of PG-degrading enzymes is the lytic transglycosylases, which produce anhydromuropeptides, a modification that caps the PG chains and contributes to bacterial virulence. Previously, it was reported that the polar-growing Gram-negative plant pathogen *Agrobacterium tumefaciens* lacks anhydromuropeptides. Here, we report the identification of an enzyme, MdaA (MurNAc deacetylase A), which specifically removes the acetyl group from anhydromuropeptide chain termini in *A. tumefaciens*, resolving this apparent anomaly. *A. tumefaciens* lacking MdaA accumulates canonical anhydromuropeptides, whereas MdaA was able to deacetylate anhydro-*N*-acetyl muramic acid in purified sacculi that lack this modification. As for other PG deacetylases, MdaA belongs to the CE4 family of carbohydrate esterases but harbors an unusual Cys residue in its active site. MdaA is conserved in other polar-growing bacteria, suggesting a possible link between PG chain terminus deacetylation and polar growth.

A defining characteristic of bacteria is the presence of a peptidoglycan (PG) cell wall, a mesh-like exoskeleton that protects them from their own internal turgor pressure and determines cell shape. PG consists of chains of alternating GlcNAc and *N*-acetylmuramic acid (MurNAc) sugars crosslinked by short peptide chains, most commonly l-Ala-d-Glu-m-DAP-d-Ala-d-Ala in Gram-negative bacteria (1). The cell wall is a highly dynamic structure, and bacteria possess an enzymatic toolkit which they use to remodel it during growth, division, and adaption to their environment. One part of this toolkit is the lytic transglycosylases (LTs), enzymes that cleave PG between the MurNAc and GlcNAc sugars with concomitant 1,6-cyclization of the MurNAc, resulting in glycan chains that terminate in a 1,6-anhydromuropeptide at the reducing end (2). LTs can be exolytic (3), where they cleave muropeptides from the end of the glycan chains releasing free anhydromuropeptides, or endolytic (4), where they cleave within the chains. Since anhydromuropeptides form the chain termini, the level of anhydromuropeptides in the cell wall can be used to determine the average length of the glycan chains.

The Gram-negative Alphaproteobacterium *Agrobacterium tumefaciens* is well known for its ability to infect plants, where it makes use of an oncogenic Ti plasmid to hijack host cells and cause crown gall disease (5). More recently, it has also gained traction as a model organism in bacterial cell biology because of its unusual characteristic of unipolar growth (6) and the abundance of potential fundamental insights into bacterial physiology that result.

It was reported that *A. tumefaciens* lacks anhydromuropeptides in its cell wall (6), suggesting that it has either another means of chain termination or extremely long glycan chains. We recently found that *A. tumefaciens* lacking its PG recycling transporter YejBEF-YepA sheds typical soluble anhydromuropeptides into the culture medium (7), while it has also been reported to have active canonical LTs (8). Here, we sought to understand the PG chain termination chemistry in *A. tumefaciens* and resolve this apparent anomaly. We report that *A. tumefaciens* has a PG deacetylase, MdaA, that specifically cleaves the acetyl group on its anhydromuropeptide chain termini, resulting in PG chains that terminate in deacetylated 1,6-anhydromuramic acid at the reducing end. As with other known PG deacetylases, MdaA belongs to the CE4 family of metal-dependent carbohydrate esterases utilizing a NodB domain for catalysis (9) but uniquely has a canonical metal-coordinating Asp residue replaced with Cys. MdaA orthologs are particularly conserved among polar-growing bacteria, many of which have been characterized as having a highly ld-crosslinked PG, suggesting a possible connection with polar growth or ld crosslinking.

## Results

### *A. tumefaciens* has deacetylated anhydromuropeptides

**Figure 1:**
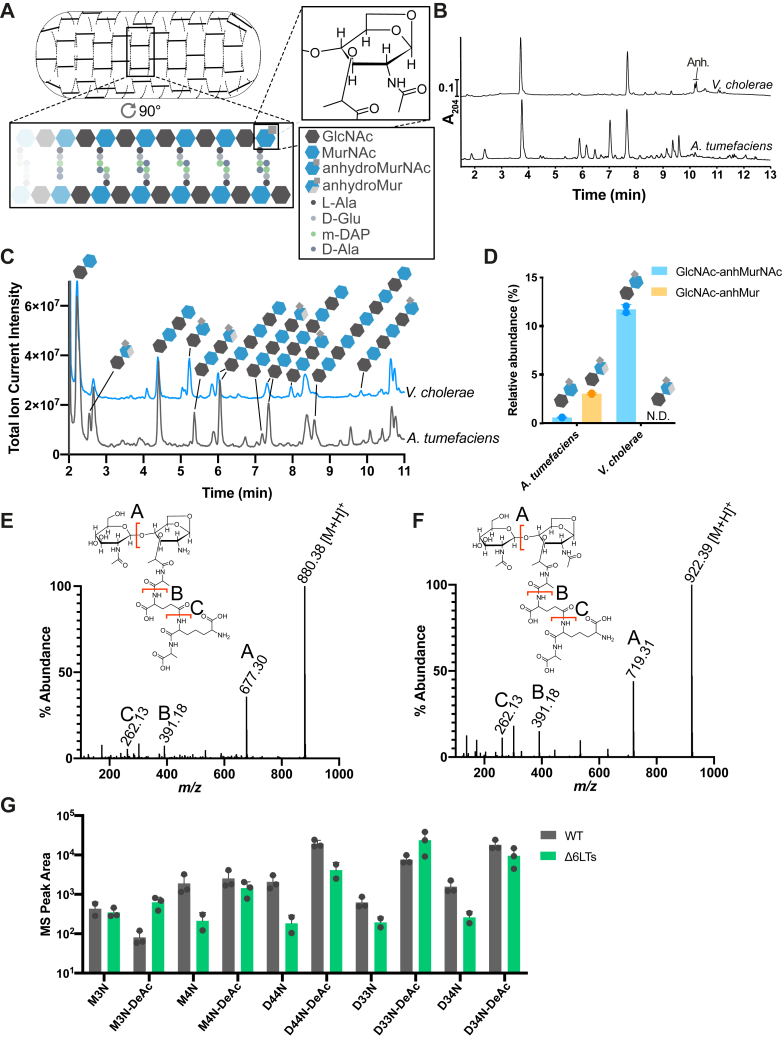
***Agrobacterium tumefaciens* has deacetylated muropeptides.***A*, schematic diagram of PG structure in Gram-negative bacteria. *B*, muropeptide profiles obtained from *Vibrio cholerae* and *A. tumefaciens*, showing apparent lack of anhydromuropeptides in *A. tumefaciens*. *C*, LC–MS traces of PG from *V. cholerae* and *A. tumefaciens* digested with amidase and muramidase enzymes. Glycan identities are outlined in Table S3. *D*, relative abundance of anhydro- and deacetylated anhydrodisaccharides in *A. tumefaciens* and *V. cholerae*. *E* and *F*, MS/MS fragmentation spectra obtained from corresponding peaks in muramidase digests of *A. tumefaciens* and *V. cholerae* PG of the its anhydroMurNAc-deacetylated tetrapeptide monomer (*A. tumefaciens*, *E*) and its acetylated version (*V. cholerae*, *F*) (*G*). Peak area of muropeptide species in the WT and Δ6LT strains determined by UPLC–MS. Peak identities as for Fig. S2*C*: D33N, l,d-crosslinked dimer of M3N and M3; D34N, l,d-crosslinked dimer of M3N and M4; D44N, d,d-crosslinked dimer of M4N and M4 (*i.e.*, not anhydrous); M3N, anhydrodisaccharide tripeptide; M4N, anhydrodisaccharide tetrapeptide. PG, peptidoglycan.

PG glycan chains typically terminate in a 1,6-anhydromuropeptide at their reducing end (Fig. 1*A*). However, PG from *A. tumefaciens* has been reported to lack anhydromuropeptides (6), and comparison of its chemical PG profile with that of the gammaproteobacterium *Vibrio cholerae* shows their apparent absence (Fig. 1*B*). To investigate the PG chain termination chemistry in *A. tumefaciens*, we digested purified PG with an amidase, which cleaves the peptide chains from the glycan chains, as well as a muramidase, allowing separation and analysis of the disaccharides. As would be expected, we observed abundant GlcNAc–MurNAc disaccharides, while small amounts of canonical anhydromuramyl disaccharides (GlcNAc–anhydroMurNAc) were also present (Fig. 1, *C* and *D* and Table S3). Interestingly, we observed disaccharides with an *m/z* ratio 62 below that expected for GlcNAc–MurNAc, which were not present in the *V. cholerae* control. MS/MS fragmentation analysis revealed that the mass loss occurred from the MurNAc sugar, since the fragment corresponding to GlcNAc(-H_2_O) at *m/z* 204.08 was intact. The loss of water during the formation of anhydroMurNAc confers a loss of *m/z* 20, whereas the further loss of *m/z* 42 suggested the loss of an acetyl group.

We then used LC–MS to look for deacetylated muropeptides in a muramidase digest of purified *A. tumefaciens* PG and identified muropeptide species with a loss of *m/z* 62. Comparison of the fragmentation pattern of the predominant monomer species (Fig. 1*E*, *m/z* 880.38) with GlcNAc–anhMurNAc-tetrapeptide from *V. cholerae* (Fig. 1*F*, *m/z* 922.39) demonstrated a loss of *m/z* 42 from the proposed MurN-peptide fragment (A, *m/z* 677.30) but not the d-Glu-m-DAP-d-Ala fragment (B, *m/z* 391.18) or GlcNAc-containing parental ion (*m/z* 880.38). This modification was not observed on any nonanhydromuropeptides. Finally, to study the effect of decreasing total anhydromuropeptides on the levels of deacetylated anhydromuropeptides, we quantified the anhydro- and deacetylated anhydromuropeptides by UPLC–MS in a Δ6 LT mutant (ΔAtu0009ΔAtu0092ΔAtu1022ΔAtu2112ΔAtu2117ΔAtu3779), which includes *mltB* (Atu3779), previously shown to be the most active in β-lactamase induction (8) indicating a significant PG turnover activity that is not compensated for by the other LTs. If the deacetylase activity uses chain termini as substrate, it would be expected that decreasing the levels of these by removing the enzymes that produce them would result in a decrease in their deacetylated versions. While the overall picture was complicated because of unrelated changes in the cell wall of the Δ6 LT mutant, we found an overall reduction of anhydromuropeptide species in the LT mutant with respect to the WT (Fig. 1*G*, structures of anhydromuropeptide species are listed in Fig. S2*C*). The relation between canonical anhydromuropeptides and their deacetylated variants was generally linear except for the deacetylated tripeptide species (M3N, anhydrodisaccharide tripeptide and D33N, its l,d-crosslinked dimer), suggesting these anhydromuropeptides are more effectively deacetylated when tetrapeptide species are not competing. In sum, we conclude that *A. tumefaciens* has a PG deacetylation activity, which is specific for the anhydroMurNAc chain termini, explaining the apparent lack of anhydromuropeptides in previous analyses.

### Identification of MdaA as the AnhMurNAc deacetylase

**Figure 2:**
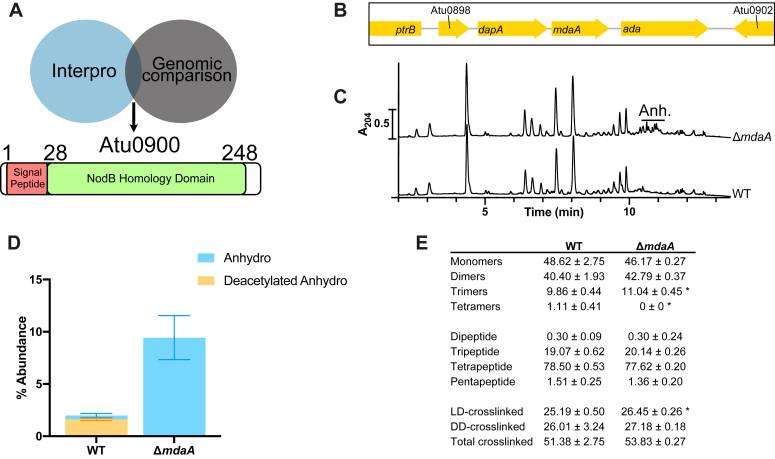
**MdaA is the anhMurNAc deacetylase.***A*, identification of Atu0900 (MdaA) as a NodB-homology domain deacetylase in the genome of *Agrobacterium tumefaciens*. Numbers correspond to amino acid positions in the peptide sequence. *B*, genomic context of MdaA. *C*, muropeptide profiles of *A. tumefaciens* WT and Δ*mdaA* strains. *D*, abundance of anhydro- and deacetylated anhydromuropeptides in PG analysis of *A. tumefaciens* WT and Δ*mdaA* strains. *E*, summary table of major PG features determined from PG analysis of *A. tumefaciens* WT and Δ*mdaA* strains. Data presented as mean ± standard deviation determined from three biological replicates. ∗ indicates statistical significance by Student’s *t* test, *p* < 0.05. PG, peptidoglycan.

We next sought to identify the enzyme responsible for this modification. PG deacetylases studied previously contain the NodB homology domain (9), which is contained in five proteins in the *A. tumefaciens C58* proteome (10). To narrow down the possibilities, we turned to previous data we obtained from a chemometric analysis of PG from other Alphaproteobacteria (11) to look for other bacteria that had or did not have deacetylated AnhMurNAc in its PG, enabling a comparative genome analysis (Fig. S1). We observed deacetylated anhydromuropeptides in PG from the magnetotactic bacterium *Magnetospirillum gryhiswaldense* and therefore used the Integrated Microbial Genomes database (12) to identify genes that were present in *A. tumefaciens* and *M. gryphiswaldense* but not the control bacteria *Sinorhizobium meliloti*, *Caulobacter crescentus*, *V. cholerae*, or *Escherichia coli*, which all lack the modification. From the list of genes unique to *A. tumefaciens* and *M. gryphiswaldense*, there was only one with a NodB homology domain, Atu0900. Atu0900 is located in a small cluster containing a DUF930-containing rhizobiales-specific protein of unknown function (Atu0898), a dihydrodipicolinate synthase (*dapA*, Atu0899), and a DNA repair methyltransferase (*ada*, Atu0901) (Fig. 2*B*). Furthermore, because of the presence of a putative signal peptide targeting it to the periplasm, Atu0900 represented a reasonable candidate for the anhMurNAc deacetylase (Fig. 2*A*).

To test whether Atu0900 is the anhMurNAc deacetylase, we deleted it and analyzed the purified PG profile from this mutant to check for peaks corresponding to canonical anhydromuropeptides. We observed a complete absence of deacetylated anhydromuropeptides in the ΔAtu0900 mutant (Figs. 2, *B* and *C* and S2), and hence, we renamed it MdaA for MurNAc deacetylase A. Remarkably, the total amount of anhydromuropeptides increased roughly fourfold, to similar (or even higher) levels observed in other species such as *E. coli* (13) suggesting that anhMurNAc deacetylation could (directly or indirectly) regulate LT activity.

### Role of MdaA

To determine any requirement of MdaA for fitness under laboratory conditions, we tested sensitivity of the Δ*mdaA* mutant to a panel of antibiotics but did not observe any increased sensitivity during growth on lysogeny broth (LB) (Fig. S3*A*). To further probe any potential fitness defects, we used high-throughput transposon sequencing (Tn-Seq) to identify genes that confer greater or reduced fitness when interrupted by transposon insertion (14). Our Tn-Seq analysis showed only weak hits in both read ratio and statistical significance (Figs. S3, *B* and *C* and supporting information 1). There were small but significant reductions in hits in the upstream region of a gene annotated as the GntR-family regulator of hexuronate transporter and metabolism *exuR* (15) (Atu3252) and an unannotated MarR-family transcriptional regulator (Atu4504).

The tRNA adenosine dimethylallytransferase MiaA (Atu2039) showed as having an increased number of Tn insertions compared with the WT strain suggesting that its interruption confers a fitness benefit. Interestingly, MiaA has previously been linked to virulence of *A. tumefaciens* (16) through the regulation of the *vir* genes and also in the production of free isopentenyladenosine, a cytokinin that influences plant cell division and differentiation (17). Other synthetically beneficial hits were a bifunctional purine biosynthesis protein PurH (Atu2823), a phosphoglucomutase ExoC (Atu4074), which is likely involved in exopolysaccharide or lipopolysaccharide production, a SPOR domain–containing d,d-carboxypeptidase DacA (Atu1505), which cleaves the C-terminal d-alanine from PG pentapeptides, Atu0439, which occurs in a region containing prophage-related genes and the uncharacterized proteins Atu0798, Atu1056, and Atu1835.

Since *A. tumefaciens* is a plant pathogen, we hypothesized that deacetylation of its anhydromuropeptides may be a strategy to avoid plant immunity. Anhydromuropeptides are well established as an immune-associated signal: the tracheal cytotoxin released by *Bordetella pertussis* is anhydromuramyltetrapeptide (M4N) (18, 19). Purified *A. tumefaciens* muropeptides have previously been shown to activate plant immune responses (20), though notably this analysis excluded anhydromuropeptides. To test for any possible virulence defect in the Δ*mdaA* mutant, we stab-inoculated *Nicotiana benthamiana* shoots with the *A. tumefaciens* WT or Δ*mdaA* strains (Fig. S3*D*). However, we did not observe any differences in tumor formation.

**Figure 3:**
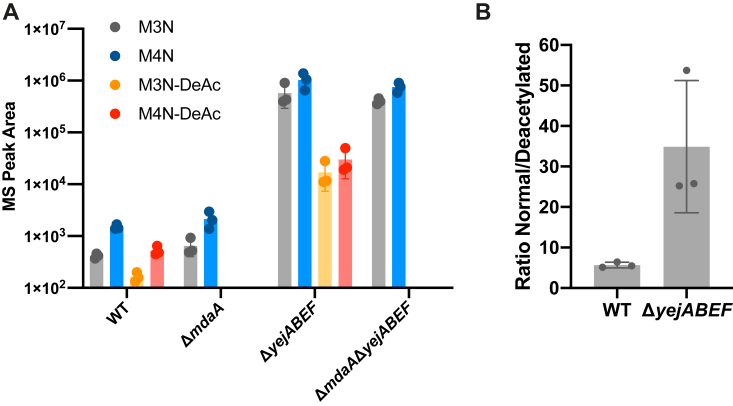
***Agrobacterium tumefaciens* sheds both normal and deacetylated muropeptides to the growth medium.***A*, mass spectrometry (MS) peak areas of integrated extracted ion chromatograms of anhydrodisaccharide tripeptide (M3N), anhydrodisaccharide tetrapeptide (M4N), and their deacetylated variants M3N-DeAc and M4N-DeAc obtained from supernatant of *A. tumefaciens* WT, Δ*mdaA*, Δ*yejABEF*, and Δ*mdaA*Δ*yejABEF* strains. *B*, ratio of peak areas of deacetylated to acetylated anhydromuropeptide species in supernatant from WT and Δ*yejABEF* strains.

*A. tumefaciens* lacks the muropeptide permease AmpG, instead using an ABC transporter YejBEF-YepA to recycle PG, and mutants in YejBEF-YepA shed anhydromuropeptides to their growth medium (7). We used LC–MS to compare the soluble muropeptide composition of the supernatant of the *A. tumefaciens* WT, Δ*mdaA*, Δ*yejABEF*, and Δ*mdaA*Δ*yejABEF* strains (Fig. 3*A*). We found that deacetylated anhydromuropeptides are released by the *A. tumefaciens* WT strain in small amounts but are more prevalent in their normal acetylated form. Interestingly, in the supernatant of the Δ*yejABEF* mutant, both acetylated and deacetylated anhydromuropeptides were released in much higher amounts, but the relative amounts of acetylated and deacetylated muropeptides were highly altered (Fig. 3*B*). There was a considerably higher ratio of acetylated to deacetylated anhydromuropeptides released compared with the WT.

### MdaA orthologs are present in other polar-growing bacteria

**Figure 4:**
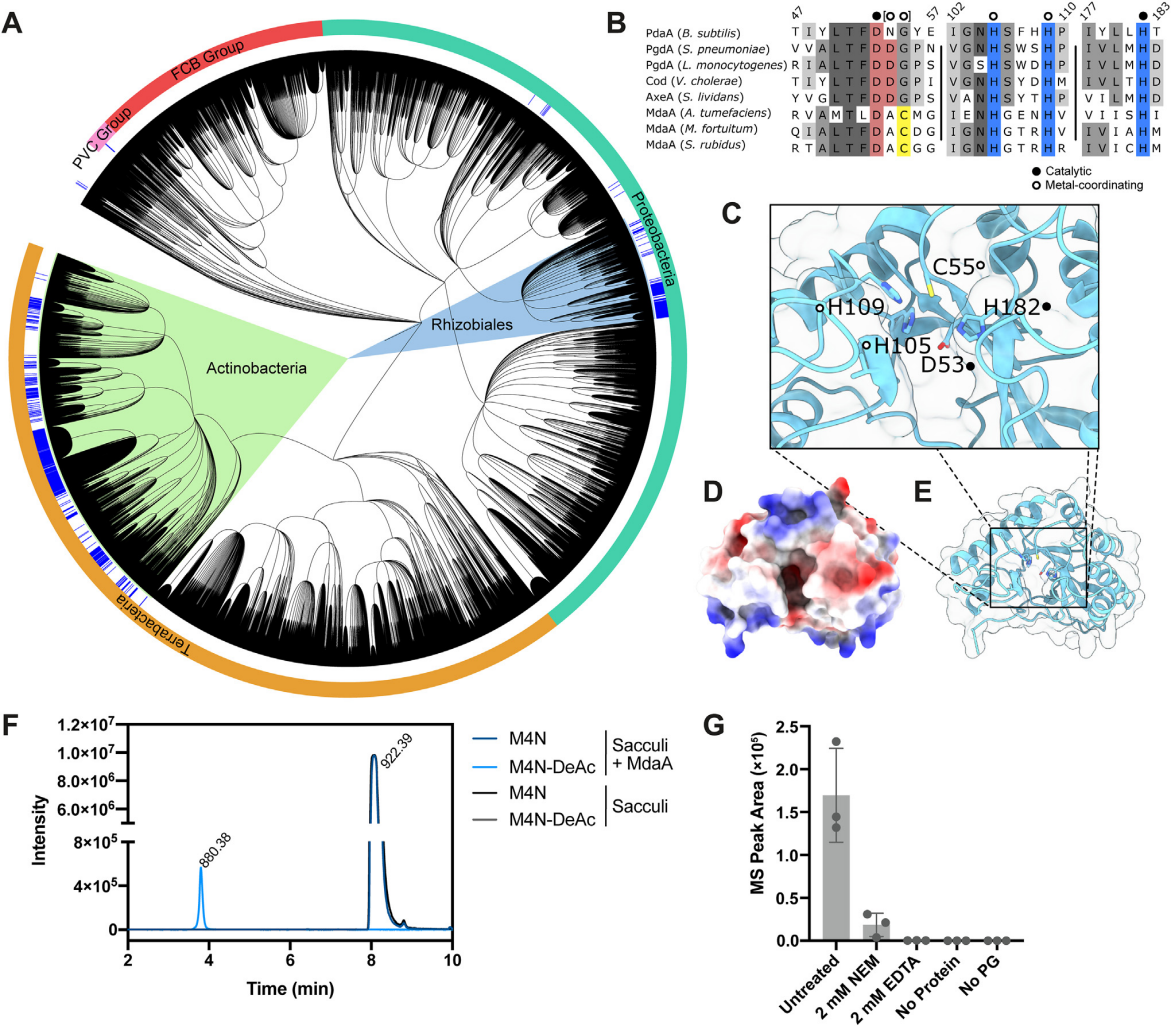
**MdaA is conserved in other bacteria.***A*, phylogenetic tree showing distribution of MdaA in bacteria. *B*, alignment of active site sequences of various CE4-family deacetylases, which are active on PG (PdaA, PgdA, and MdaA), chitin (cod), and acetylxylan (AxeA), indicating metal-coordinating and catalytic residues; amino acid positions are indicated at the *top*. Species: *Bacillus subtilis* (PdaA), *Streptococcus pneumoniae* (PgdA), *Listeria monocytogenes* (PgdA), *Vibrio cholerae* (cod), *Streptomyces lividans* (AxeA), *Agrobacterium tumefaciens* (MdaA), *Mycolicibacter fortuitum* (MdaA), and *Streptomyces rubidus* (MdaA). *C*–*E*, AlphaFold2 model of MdaA lacking signal peptide, displayed using ChimeraX. *C*, view of active site showing arrangement of proposed catalytic and metal-coordinating residues. *D*, surface map with calculated electrostatic charges indicated in *blue* (positive) and *red* (negative). *E*, *cartoon representation* of MdaA. *F*, extracted ion chromatograms (EICs) of ion masses corresponding to M4N (*m/z* 922.39) and M4N-DeAc (*m/z* 880.38) obtained from LC–MS analysis of lytic transglycosylase–treated *V. cholerae* sacculi, with and without MdaA treatment. *G*, EIC peak areas of M4N-DeAc obtained from MdaA-treated sacculi with addition of 2 mM *N*-ethylmaleimide (NEM), 2 mM EDTA, or control reactions with no protein or no PG added. EIC, extracted ion chromatogram; PG, peptidoglycan.

To better understand the biological role of MdaA-mediated PG deacetylation in *A. tumefaciens*, we used OrthoDB, version 11 (21) to look for orthologous proteins in other species, which might give some hint as to its function. Orthologs of MdaA mapped to two major orders of bacteria: the Gram-negative *A. tumefaciens*-containing Rhizobiales and the Gram-positive Actinomycetales including some Corynebacterium, *Streptomyces*, and Mycolicibacterium species (Fig. 4*A*). Although we have not tested whether these constitute *bona fide* MdaA orthologs, we note that MurNAc deacetylation has previously been observed in *Streptomyces coelicolour* (22). In addition, these enzymes contain some sequence peculiarities that are shared by MdaA but not other characterized CE4 family deacetylases (Fig. 4*B*). Most strikingly, a metal-coordinating Asp residue (23, 24) has been replaced with Cys in MdaA (C55).

Unlike the Ser-dependent penicillin-binding proteins, ld-transpeptidase enzymes (LDTs) use a Cys for catalysis (25), which is vulnerable to oxidation in the periplasm (26). Since C55 of MdaA is also a lone cysteine that is predicted to be surface exposed (Fig. 4, *C*–*E*), located in the active site, and MdaA is also taxonomically associated with LDT-using species, we hypothesized that it may play some role in protection against oxidative stress by acting as a redox switch or a sensor (27, 28). We expressed MdaA lacking its first 20 amino acids (*i.e.*, signal peptide) in the cytoplasm of *E. coli* and purified it. Initially, we were unable to obtain MdaA that was active on pure, fully acetylated, PG sacculi. However, when we purified MdaA under reducing conditions (1 mM DTT in all buffers), we were able to detect the production of M4N-DeAc on PG sacculi *in vitro* (Fig. 4*F*) under slightly acidic conditions (pH 6.3). To investigate the requirement of the C55 residue for catalysis, we added 2 mM *N*-ethylmaleimide (NEM) to the reaction mixture, which binds irreversibly to sulphydryl groups (29). A corresponding decrease in anhMurNAc deacetylation of the *V. cholerae* sacculi was observed with NEM addition (Fig. 4*G*), with the little remaining activity probably attributable to DTT remaining in the reaction mixture sequestering the NEM. Interestingly, addition of 2 mM EDTA resulted in a total loss of MdaA activity, suggesting that it does require a metal ion cofactor as for other CE4-family esterases.

Although the Rhizobiales and Actinomycetales seem distant relatives, they share an important feature: polar growth, with a higher proportion of PG crosslinking mediated by LDTs compared with laterally growing species (30, 31). To test for any polar or sidewall localization, which might contribute to polar growth, we wanted to localize MdaA-sfGFP in WT *A. tumefaciens* labeled with the fluorescent d-amino acid HCC amino-d-alanine (HADA) (32) to visualize the active locations of PG synthesis.

To express plasmid-encoded MdaA in the *A. tumefaciens*, we modified pSRKKm (33) promoters by replacing *lacI*^*q*^ with the gene encoding the cumate responsive repressor CymR (34, 35) and replacing the *lacO* operator sites with *cuO* operator sites. Expression from P_cym_ requires the presence of cumate and is slightly higher than expression from IPTG-inducible promoters such as P_lac_, enabling both compatibility with IPTG-inducible depletion strains and sufficient levels of MdaA-sfGFP expression for microscopy analysis. Although higher concentrations of cumate inhibit growth of WT *A. tumefaciens*, concentrations of less than 0.5 mM do not impair growth of WT cells.

Expression of MdaA-sfGFP incorporating its native signal peptide from the cumate-inducible promoter did not have a significant impact on the distribution of cell length in WT cells or Δ*mdaA* cells (Fig. S4*A*, indicating expression of MdaA-sfGFP does not impair proper cell morphology. Localization of MdaA-sfGFP appeared dispersed, yet patchy in WT and Δ*mdaA* cells. Patches of MdaA-sfGFP appeared to be randomly distributed throughout the cell and did not colocalize with the growth poles, as determined by HADA labeling (Fig. S4*B*). To explore the MdaA-sfGFP localization pattern at the population level, demographs were constructed and illustrate the unevenly dispersed localization pattern of MdaA-sfGFP (Fig. S4*C*). Many cells have more intense GFP signal concentrated in a particular subcellular localization, suggesting that MdaA-sfGFP may have a dynamic localization pattern. Indeed, time-lapse microscopy reveals that MdaA-sfGFP is highly dynamic and rapidly moves throughout the cell (Movies S1 and S2; Fig. S4*D*). To further confirm that MdaA-sfGFP is not concentrated at the growth pole, we introduced MdaA-sfGFP into an *ftsZ* depletion strain (36). Depletion of *ftsZ* causes growth poles to accumulate, and some proteins involved in polar growth become trapped at growth poles. During depletion of *ftsZ*, MdaA-sfGFP remains dispersed throughout the entire cell (Fig. S4*E*).

## Discussion

Numerous modifications of the PG sugars have been reported in a range of bacteria and typically constitute an adaption to a particular niche or stress (37). Here, we report a specific *N*-deacetylation of anhMurNAc residues occurring at the PG chain termini of *A. tumefaciens* PG, which is catalyzed by a novel anhMurNAc deacetylase, MdaA. All PG *N*-deacetylases characterized so far have been GlcNAc specific, with the exception of PdaA from *Bacillus subtilis*, which deacetylates MurNAc during the production of muramic δ-lactam (38, 39, 40, 41).

*A. tumefaciens* is well known for its ability to invade plants and produce crown galls. Since PG is highly immunogenic, we hypothesized that deacetylation of anhydroMurNAc may play some role in avoiding plant immunity. However, *A. tumefaciens* primarily sheds normal anhydromuropeptides to its growth medium, suggesting that this may not be the case. Supporting a potential role in pathogenesis, *mdaA* displayed a roughly 11-fold upregulation in transcriptomics done in the plant tumor (42). However, Tn-Seq experiments in various plant infection–associated niches did not show any loss of fitness for MdaA insertion mutants, indicating no requirement for fitness under these conditions (43). Furthermore, our *N. benthamiana* infection experiment showed no clear difference in the ability of the WT and Δ*mdaA* strains in their ability to cause tumor formation in this model. Interestingly, we found that the ratio of acetylated to deacetylated anhydromuropeptides shed to the medium by a Δ*yejABEF* mutant was considerably increased, suggesting that *A. tumefaciens* could preferentially recycle normal anhydromuropeptides over deacetylated. This could indicate a strategy where the less immunogenic deacetylated muropeptides are released as a signal. Other possible roles of MdaA in supporting virulence exist. For example, a PG deacetylase is involved in virulence of *Legionella pneumophila* (41), where it has a dual function in both lysozyme resistance and supporting the localization of the Dot/Icm type IV secretion system. *A. tumefaciens* also utilizes a type IV secretion system for virulence, raising the possibility that MurNAc deacetylation could play a similar role in *A. tumefaciens*.

The conservation of MdaA in the distantly related Rhizobiales and Actinomycetales is intriguing, because of the shared polar-growth mode and use of LDTs by these species. The active site of MdaA is unique among characterized CE4-family PG deacetylases in containing a Cys residue in place of the canonical metal-coordinating Asp. The acid–base-nucleophile catalytic triad is a common feature of lytic enzymes. In CE4-family esterases, the nucleophile is typically water activated by the Asp-His-His-coordinated metal ion (23). It would be tempting to speculate that C55 of MdaA could substitute as nucleophile for the deacetylation reaction, in a manner reminiscent of Cys-Asp-His catalytic triads of some cysteine proteases (44), and notably also the ld-transpeptidases (45). However, that EDTA inhibited MdaA activity *in vitro* suggests that MdaA relies on a metal ion for catalysis, as shown for other CE4-family esterases. In addition, the two His residues that also typically coordinate the metal ion are conserved in MdaA (His105 and His109, Fig. 4*B*). Cysteine residues are able to bind metal ions with a particular affinity for Zinc (46), and the inhibition of MdaA by NEM indicates that the thiol group of C55 is required for activity.

The significant overall increase in anhydromuropeptides seen in the Δ*mdaA* mutant (Fig. 2*D*) raises the possibility that anhMurNAc deacetylation may regulate the activity of LTs. The lack of a significant fitness cost associated with *mdaA* deletion suggests that this is not a significant factor in its own growth or morphology. However, many phages or competing bacteria use LTs to degrade their prey’s cell wall and cause them to lyse, and thus deacetylation of chain termini could be a mechanism of protection against LTs used as weapons by other organisms. The role of MdaA-mediated anhMurNAc deacetylation in interspecies interactions is therefore an interesting topic for further study.

## Experimental procedures

### Strains and growth conditions

Strains used in this study are listed in Table S1. *E. coli* strains were grown in LB or LB agar at 37 °C. *A. tumefaciens* was grown in LB or *A. tumefaciens* glucose and (NH_4_)_2_SO_4_/*A. tumefaciens* sucrose and (NH_4_)_2_SO_4_ minimal medium prepared as described (47) at 30 °C. Kanamycin was added to media for selection as required at concentrations of 50 μg/ml for *E. coli* and 300 μg/ml for *A. tumefaciens*.

### PG isolation and analysis

PG isolation was carried out as described previously (48). Briefly, cells from 10 ml overnight stationary phase cultures were pelleted at 5000*g* and resuspended in 5 ml PBS, added an equal volume of boiling 10% SDS and left boiling for 2 h with stirring, then stirred overnight at room temperature. The remaining insoluble PG was pelleted by ultracentrifugation at ∼120,000*g* for 15 min in an Optima MAX-TL ultracentrifuge and washed three times in water to remove the SDS. The final pellet was resuspended in 100 μl of 50 mM sodium phosphate buffer (pH 4.9) and digested overnight at 37 °C with muramidase at a final concentration of 30 μg ml^−1^. Digestion was stopped by boiling the samples for 5 min, and the precipitated protein was removed by centrifugation at maximum speed in a benchtop centrifuge. The supernatants were adjusted to pH 8 to 9 using 0.5 M borate buffer (pH 9.5), and reduction was carried out by addition of sodium borohydride (final concentration of 10 mg ml^−1^) followed by incubation at room temperature for 20 min. The digested and reduced PG samples were adjusted to pH 3.5 using orthophosphoric acid as preparation for chromatographic analysis.

Chromatographic separation of the muropeptides was carried out using an Acquity H-Class UPLC (Waters) equipped with an Acquity BEH C18 column (130 Å pore size, 1.7 μm particle size, 2.1 mm by 150 mm column size) maintained at 45 °C. Separation was carried out using a linear gradient from 0.1% formic acid in water to 0.1% formic acid in acetonitrile over 18 min with 0.25 ml min^−1^ flow rate. UV detection was carried out at 204 nm. Muropeptides were identified using LC–MS with chromatographic separation as described, coupled to a Xevo G2-XS QTOF mass spectrometer. The QTOF instrument was operated in positive ion mode with data collection performed in the untargeted MS^e^ mode. The parameters were set as follows: capillary voltage 3.0 kV, source temperature 120 °C, desolvation temperature 350 °C, sample cone voltage 40 V, cone gas flow 100 l h^−1^, and desolvation gas flow 500 l h^−1^. Data acquisition and processing was done using the MassLynx software (Waters); a compound library of muropeptides was built by drawing the structures in ChemSketch (http://www.acdlabs.com) to facilitate peak identification.

### PG amidase digestion

The gene encoding the amidase AmpDH3 from *Pseudomonas aeruginosa* PAO1 was cloned onto pET28b (Novagen) with a C-terminal hexa-His tag for production in *E. coli BL21*(DE3) cells. Expression was induced at an absorbance of 0.4 at 600 nm with 1 mM IPTG overnight at room temperature. Cultures were pelleted, and cell pellets were resuspended in 100 mM Tris–HCl (pH 8) and 50 mM NaCl with cOmplete Protease Inhibitor Cocktail Tablets (Roche) added following the manufacturer’s instructions. Cells were lysed with two passes through a French press at 1.5 bar. The insoluble fraction was separated by centrifugation (30 min, 50,000 rpm). The pellet was stirred overnight in 100 mM Tris–HCl (pH 8), 50 mM NaCl supplemented with 0.1% Triton at 4 °C, and centrifuged for 30 min at 75,000*g*. The lysate was added to nickel–nitrilotriacetic acid agarose columns (Qiagen) and eluted with a discontinuous imidazole gradient using an ÄKTA Go system. Protein purity was visualized by SDS-PAGE.

*A. tumefaciens* and *V. cholerae* sacculi were digested overnight with muramidase (80 μg/ml) and heat inactivated. Amidase assays were done with modified protocol version of a previously published protocol (49). Briefly, a reaction mixture consisting of 50 μl sacculi and 1 μM AmpDh3 in 20 mM Tris–HCl (pH 6.8), 30 mM NaCl, and 10 mM MgCl_2_ was incubated overnight at 37 °C with shaking. Reactions were heat inactivated at 100 °C for 5 min and centrifuged for 10 min at 21,000*g* to pellet the insoluble material. The supernatant was reduced and prepared for chromatographic injection as described above.

### Supernatant soluble muropeptide analysis

Cultures of *A. tumefaciens* were centrifuged at 10,000*g* for 2 min to pellet the cells, and the supernatants were decanted and filtered through a 0.22 μm pore-size filter to remove any remaining cells. The supernatants were boiled for 5 min to precipitate any secreted proteins, centrifuged at ∼21,000*g* for 15 min to remove insoluble material, and filtered into UPLC vials for LC–MS analysis as described previously.

### Bioinformatic analyses

Orthologs of MdaA in other bacterial species were identified using OrthoDB, version 11 (group 9814083at2) (21). PhyloT v2 (Biobyte) was used to produce a phylogenetic tree of all species on OrthoDB, and the MdaA orthologs were mapped against this using iTOL (50). Selected MdaA ortholog sequences from *A. tumefaciens*, *Mycolicibacter fortuitum*, and *Streptomyces rubidus* were aligned with other CE4-family esterases using Muscle v5 (51) for comparison, with Unipro UGENE (52) used for sequence alignment visualization. The 3D structure of MdaA from *A. tumefaciens* was predicted using the ColabFold (version 1.5.2) (53) interface to AlphaFold2 (54), with MMSeqs2 (55) used for sequence clustering. The *mdaA* gene cluster in *A. tumefaciens* was visualized using Gene Graphics (56).

### Protein purification and *in vitro* reactions

MdaA from *A. tumefaciens* C58 lacking its first 20 amino acids was cloned into the pET28b plasmid (Novagen) in *E. coli* DH5ɑ and transformed into *E. coli* BL21 for expression. Expression was done overnight in 2 l Terrific broth (57) at 16 °C with addition of 0.1 mM IPTG for induction. The cells were pelleted at 6000*g* for 15 min, resuspended in PBS with 1 mM DTT, and then broken using three passes through a French press at 1.5 bar. The cell lysate was clarified by centrifugation at 75,000*g* for 50 min, and the soluble fraction was used for purification. Purification was done using an ÄKTA Go FPLC system (Cytiva) in three stages. First, nickel-affinity chromatography was done using a HisTrap FF 5 ml column (Cytiva) in PBS with 1 mM DTT and a gradient from 20 to 500 mM imidazole for elution. Then, size-exclusion chromatography was done using a Superdex 200 Increase 10/300 GL column with PBS with 1 mM DTT as buffer. Finally, cation exchange chromatography was used to remove remaining contaminants using a HiTrap Capto SP ImpRes 1 ml cation exchange column using 10 mM Tris–HCl (pH 6.3) and 1 mM DTT as buffer and a gradient from 0 to 1 M NaCl for elution. A Vivaspin 20 centrifugal concentrator (10 kDa molecular weight cutoff; Sigma) was used to concentrate the protein and exchange the buffer to 10 mM Tris–HCl (pH 6.3) and 1 mM DTT for *in vitro* reactions. The concentration of purified MdaA was determined using the Bradford protein assay reagent (Bio-Rad) with dilutions of bovine serum albumin (Thermo) as standard.

*In vitro* deacetylation assays were done on sacculi purified from *V. cholerae* N16961, which does not have deacetylated muropeptides in its PG (58). About 5 μg MdaA was added to *V. cholerae* sacculi in a final reaction volume of 50 μl in a reaction buffer containing 100 mM Tris–HCl (pH 6.3), ∼0.5 mM DTT, and 100 mM NaCl. As control, identical reactions were set up with either no protein or no PG added. The reactions were incubated overnight at 30 °C with shaking, and the next day, 10 μg of a purified LT (MltA) was added, and the samples were incubated at 37 °C with shaking for 4 h, to digest the sacculi into soluble anhydromuropeptides and facilitate LC–MS analysis, which was carried out as described previously.

### Antibiotic sensitivity testing

Sensitivity of the *A. tumefaciens* WT and Δ*mdaA* strains to various antibiotics was tested on agar plates using MIC (minimum inhibitory concentration) test strips (Liofilchem). Exponentially growing cultures of *A. tumefaciens* strains were adjusted to an absorbance of 0.1 at 600 nm in PBS before being spread across LB plates using a sterile cotton swab three times; the plate was rotated 60° between each spreading. The strip was applied once the plate surface had dried fully.

### *N. benthamiana* infection experiments

Inoculation of *N. benthamiana* shoots with *A. tumefaciens* WT and Δ*mdaA* strains was done as described elsewhere (59).

### Cumate-inducible vector construction

All strains and plasmids used are listed in Table S1, whereas primers used are listed in Table S2. To construct pSRKKm-P_cym_, a synthesized gBlock from Integrated DNA Technologies was made containing the regulatory elements of the cumate system similar to previously described plasmid constructs (34, 35). The sequence encoding the cumate repressor was codon optimized for *A. tumefaciens* and placed under the control of the constitutive kanamycin promoter from pSRKKm-P_lac_-sf*gfp*. The synthesized gBlock was digested overnight with EcoRI and NdeI. The resulting fragment was then ligated into cut pSRKKm-P_lac_-sf*gfp*, thereby replacing the original *lac* promoter and repressor with the cumate repressor and cumate-regulated promoter. To construct expression vector containing *mdaA-sfGFP*, the coding sequence was amplified from purified C58 genomic DNA using primers indicated in Table S2. The amplicons were digested overnight with NdeI and BamHI and ligated into cut pSRKKm-P_cym_-*sfgfp* using NEB T4 DNA ligase at 4 °C overnight. All vectors were verified by sequencing and introduced into *A. tumefaciens* strains utilizing standard electroporation protocols (60).

### Phase contrast and fluorescence microscopy

Exponentially growing cells (absorbance at 600 nm = ∼0.6) were spotted on 1% agarose *A. tumefaciens* glucose and (NH_4_)_2_SO_4_ pads as previously described (9) prior to imaging with an inverted Nikon Eclipse TiE with a QImaging Rolera EM-C2 1K EMCCD camera and Nikon Elements Imaging Software. Plasmid-encoded *mdaA-sfGFP* was induced by the presence of 0.2 mM cumate for 2 h prior to imaging for quantitative image analysis. Phase contrast images were used for cell length analysis using the MicrobeJ software (61). Demographs were constructed using GFP channel profiles taken along the medial axis for each cell, which were then aligned by cell length using MicrobeJ. To visualize sites of active PG synthesis, 1 ml of exponentially growing cells was labeled with the fluorescent d-amino acid, HADA, as previously described (32, 62).

### Tn-Seq

Tn-Seq was done as described elsewhere (63). Briefly, 3 to 5 × 10^5^ transposon mutants were generated per library by conjugation of *A. tumefaciens* strains with *E. coli* SM10 λ-pir carrying the Mariner transposon donor plasmid pSC189 (64) as described (60). Three independently prepared mutant libraries were prepared and sequenced per condition (*i.e.*, *A. tumefaciens* WT and Δ*mdaA*). Mutant libraries were grown in triplicate with 500 μg/ml kanamycin and 25 μg/ml streptomycin to select for the transposon and remove the donor *E. coli*. The libraries were collected, genomic DNA isolated, and pooled fragments were sequenced using a MiSeq system (Illumina). Sequenced fragments were trimmed using Cutadapt, version 2.1 (65), aligned to the *A. tumefaciens* C58 genome using Bowtie2, version 2.4.1 (66), and statistical analysis of transposon insertion sites was done using the Con-ARTIST pipeline (67).

## Data availability

All data are included in this article or the supporting information.

## Supporting information

This article contains supporting information (7, 36).

## Conflict of interest

The authors declare that they have no conflicts of interest with the contents of this article.
