# Supplementary material for: A peptidoglycan N-deacetylase specific for anhydroMurNAc chain termini in Agrobacterium tumefaciens
Source: J Biol Chem. 2023 Dec 28;300(2):105611. doi: 10.1016/j.jbc.2023.105611 (PMC10838918; doi:10.1016/j.jbc.2023.105611)
Supplement: Supporting Information and Tables [file mmc4.docx]

**Supporting information**

**A peptidoglycan *N*-deacetylase specific for anhydroMurNAc chain termini in *Agrobacterium tumefaciens***

Michael C. Gilmore^1^*, Akhilesh K. Yadav^1,2^*, Akbar Espaillat^1,5^*, Andrea A. Gust^3^, Michelle A. Williams^4^, Pamela J. B. Brown^4^, Felipe Cava^1^†

^1^ Department of Molecular Biology and Laboratory for Molecular Infection Medicine Sweden, Umeå Centre for Microbial Research, SciLifeLab, Umeå University, Umeå, Sweden.

^2^ Academy of Scientific and Innovative Research (AcSIR), Ghaziabad, 201002, Uttar Pradesh, India; Regulatory Toxicology Group, CSIR-Indian Institute of Toxicology Research (CSIR-IITR), Lucknow, 226001, Uttar Pradesh, India

^3^ Department of Plant Biochemistry, Center of Plant Molecular Biology (ZMBP), Eberhard-Karls-University of Tübingen, Tübingen, Germany

^4^ Division of Biological Sciences, University of Missouri-Columbia, Columbia, Missouri, USA

^5^ Current address: Chr. Hansen A/S, Microbial Physiology, R&D, 2970 Hoersholm, Denmark.

* These authors contributed equally

† for correspondence: [felipe.cava@umu.se](mailto:felipe.cava@umu.se)

Running title: Peptidoglycan deacetylase specific for anhydroMurNAc

Key words: Peptidoglycan, deacetylase, anhydromuropeptide, lytic transglycosylase, *Agrobacterium tumefaciens*

**Legends to Supplementary figures**

**Figure S1.** Distribution of deacetylated GlcNAc-Mur-tetrapeptide (M4N-DeAc) in a selection of Alphaproteobacteria.

**Figure S2.** (A). LC-MS traces of PG from *A. tumefaciens* WT and Δ*mdaA* digested with amidase and muramidase enzymes. (B). Muropeptide profiles obtained from PG analysis of *A. tumefaciens* WT and Δ*mdaA* strains with identified peaks indicated. (C). Table of identified peaks, proposed structures and relative abundances between WT and Δ*mdaA* strains. Abundance data represented as mean ± standard deviation. * indicates statistical significance by T-test, *p* < 0.05.

**Figure S3.** (A). Minimal inhibitory concentration values (MICs, μg/mL) of WT and Δ*mdaA* strains for a selection of antibiotics determined using E-test strips. (B). Volcano plot showing the ratio of Tn-Seq reads mapped to genes in Δ*mdaA* strain compared to WT control, plotted against inverse *p* value determined by Mann-Whitney U Test (cutoff *p*^-1^ > 20). (C). Table showing hits which pass statistical significant cutoff (*p* < 0.05) with UniProt annotations. (D). Tumours formed on shoots of *Nicotiana benthamiana* stab-inoculated with WT and Δ*mdaA* strains. Pictures taken 23 days post-inoculation.

**Figure S4.** Localization and morphology of MdaA-sfGFP expressing cells. A) Cell length analysis of WT and Δ*mdaA* cells containing pSRKKm-Pcym*-mdaA-sfGFP*. In the absence of cumate, *mdaA-sfGFP* remains uninduced (grey). In the presence of 0.2 mM cumate *mdaA-sfGFP* is induced (white). Median cell length (µm) is indicated for each strain. n = ≥ 338. B) Strains from (A) were induced with 0.2mM cumate for 2 hours then stained with HADA and ethanol fixed prior to imaging. C) Demographs depict localization of MdaA-sfGFP at a population level. Median profiles of the GFP channel of 400 cells per strain are stacked and ordered by cell length. D) Time-lapse microscopy of MdaA-sfGFP in WT and Δ*mdaA* reveals dispersed, patchy localization. Cells were induced with 0.2mM cumate for 2 hours then spotted on an agarose pad and imaged every 10 minutes. E) Localization of MdaA-sfGFP remains dispersed during depletion of the cell division protein FtsZ. The single chromosomal copy of *ftsZ* is under the control of the *tac* promoter and depletion occurs in the absence of IPTG. Cells were washed 3 times to rid the cells of IPTG then treated with 0.2 mM cumate to induce *mdaA-sfGFP* expression and incubated for 5 hours before being spotted on an ATGN agarose pad and imaged. Times represent the number of hours cells were grown without IPTG. Scale bars = 2 µm

**Legends to Supplementary Movies**

**Movie S1.** Localization of MdaA-sfGFP in WT cells. Phase contrast imaging of cells induced with 0.2 mM cumate and grown in liquid ATGN for 2 hours before spotting on an ATGN agarose pad. Images were acquired every ten minutes and movie is played at 4 frames per second for a total of 19 frames.

**Movie S2.** Localization of MdaA-sfGFP in WT cells. Fluorescence imaging of cells induced with 0.2 mM cumate and grown in liquid ATGN for 2 hours before spotting on an ATGN agarose pad. Images were acquired every ten minutes and movie is played at 4 frames per second for a total of 19 frames.

## **Legend to Supporting Data**

## **Supporting Data 1**: Tn-Seq analysis for *A. tumefaciens* Δ*mdaA* mutant compared to WT.

# Supporting Information

## **Table S1: Strains**

| **Bacterial Strain** | **FC Strain Identifier** | **Source** |
| --- | --- | --- |
| *Agrobacterium tumefaciens* C58 | FC1701 | - |
| *Escherichia coli* DH5ɑ pNPTS139 | FC2289 | Courtesy of P. Brown |
| *Agrobacterium tumefaciens* C58 Δ*mdaA* | FC2643 | This study |
| *Agrobacterium tumefaciens* C58 Δ*yejABEF* | FC3400 | Gilmore and Cava 2022 |
| *Agrobacterium tumefaciens* C58 Δ*mdaA*Δ*yejABEF* | FC3578 | This study |
| *Agrobacterium tumefaciens* C58 ΔAtu3779 | FC2487 | This study |
| *Agrobacterium tumefaciens* C58 ΔAtu0009ΔAtu0092ΔAtu1022 ΔAtu2112ΔAtu2117ΔAtu3779 | FC2605 | This study |
| *Escherichia coli* BL21 pET28b::*mdaA*-ΔSP | FC2668 | This study |
| *Escherichia coli* DH5ɑ pSRKKm-Pcym*-atu0900-sfGFP* | - | This study |
| *Agrobacterium tumefaciens* C58 pSRKKm-Pcym*-atu0900-sfGFP* | - | This study |
| *Agrobacterium tumefaciens* C58 ∆*tetRA*::mini-Tn7-GM-Ptac-*ftsZ_AT_* pSRK_Km_-Pcym*-atu0900-sfGFP* | - | Howell et al. 2019, this study |
|  |  |  |
|  |  |  |

## **Table S2: Primers**

| **Primer** | **FCP Identifier** | **Sequence (5’ -> 3’)** |
| --- | --- | --- |
| ΔAtu0900 Upstream Forward (HindIII) | FCP2633 | aaaaagcttatatgtatctcacgcgtgagg |
| ΔAtu0900 Upstream Reverse | FCP2567 | tttccgcagtgtcatgcagtgtattccc |
| ΔAtu0900 Downstream Forward | FCP2568 | ACTGCGGAAAaaacggggacagatctttcctcattcc |
| ΔAtu0900 Downstream Reverse (PstI) | FCP2569 | aaactgcagattcgaggagatgcagggc |
| pET28b::PA14_53820 Forward (NcoI) | FCP1111 | AAACCATGGTGACCATCGACTACAACAGC |
| pET28b::PA14_53820 Reverse (HindIII) | FCP1112 | AAAAAGCTTGGCCGGGTATTTCTCGTTCAGAGCG |
| pET28b::*mdaA*-ΔSP Forward (NdeI) | FCP2777 | aaacatatgctgaaaatgacgccgcagcatgacgg |
| pET28b::*mdaA*-ΔSP Reverse (HindIII) | FCP2571 | aaaaagctttcaattccccggtggcgcgaagg |
| ΔAtu2112 Upstream Forward (EcoRI) | FCP2003 | AAAGAATTCATATCGTCCTCGGTTTCCGC |
| ΔAtu2112 Upstream Reverse | FCP2004 | ATCGATCATCTGCGAAATTGCG |
| ΔAtu2112 Downstream Forward | FCP2005 | GATGATCGATATGACAGAAACAGTGAAATGGC |
| ΔAtu2112 Downstream Reverse (HindIII) | FCP2006 | AAAAAGCTTTATTTCGTCTTCGAGGATGGG |
| ΔAtu2117 Upstream Forward (EcoRI) | FCP1997 | AAAGAATTCAAGAGAATGTCTGGACAGGCGTGGC |
| ΔAtu2117 Upstream Reverse | FCP1998 | GCATTTTCAATTGCCGCCTTCGTTATGCC |
| ΔAtu2117 Downstream Forward | FCP1999 | TTGAAAATGCTGCGGCTCCC |
| ΔAtu2117 Downstream Reverse (HindIII) | FCP2000 | AAAAAGCTTTAGATGTTCCTGTCGAACACCG |
| ΔAtu0009 Upstream Forward (HindIII) | FCP2009 | AAAAAGCTTAACGCATCTTCTAGCCTTGCG |
| ΔAtu0009 Upstream Reverse | FCP2010 | TATTCATTGCTCGGATTCGG |
| ΔAtu0009 Downstream Forward | FCP2011 | GCAATGAATATATCGGCGATGAAAGGC |
| ΔAtu0009 Downstream Reverse (BamHI) | FCP2012 | AAAGGATCCGAAAGAACAATTCCTCCGC |
| ΔAtu1022 Upstream Forward (EcoRI) | FCP2015 | AAAGAATTCAATACGCTCTTCAACTCCATCCG |
| ΔAtu1022 Upstream Reverse | FCP2016 | TGGGGATTATTCAGGCACGGGCTAGCC |
| ΔAtu1022 Downstream Forward | FCP2017 | AATCCCCAGACTGTCTTTTTCATGCCG |
| ΔAtu1022 Downstream Reverse (HindIII) | FCP2018 | AAAAAGCTTTTATGCGCTTTGACCAGCGCACCC |
| ΔAtu3779 Upstream Forward (PstI) | FCP2021 | AAACTGCAGTAGAAATTCGACGGCGCCG |
| ΔAtu3779 Upstream Reverse | FCP2022 | TTTCGATTGCGAAAACGCATCGGGCG |
| ΔAtu3779 Downstream Forward | FCP2023 | GCAATCGAAATAATGTGCCGGCGAATTCGG |
| ΔAtu3779 Downstream Reverse (BamHI) | FCP2024 | AAAGGATCCTTGGCCGTTCATGTCGTAGCC |
| ΔAtu2122 Upstream Forward (EcoRI) | FCP2027 | AAAGAATTCAATCATCAGGGTTCCAATGCGG |
| ΔAtu2122 Upstream Reverse | FCP2028 | TAGTGCGATTTTCCTCGATAGGTTGTTGGC |
| ΔAtu2122 Downstream Forward | FCP2029 | ATCGCACTACCGGGCCTTTAATCTATCGG |
| ΔAtu2122 Downstream Reverse (HindIII) | FCP2030 | AAAAAGCTTATAATGACGTCTTTGAACGC |
| ΔAtu0092 Upstream Forward (PstI) | FCP2033 | AAACTGCAGATTCTTGCCCTGATGCCCATTGTCGC |
| ΔAtu0092 Upstream Reverse | FCP2034 | AGACCGAATATCGTCTTTTAATGCTGGTCGG |
| ΔAtu0092 Downstream Forward | FCP2035 | ATTCGGTCTCCTCTTGGATGG |
| ΔAtu0092 Downstream Reverse (BamHI) | FCP2036 | AAAGGATCCAAGTCGAGATCGACTGAGCCC |
| Atu0900-sfGFP Forward (NdeI) | - | CAGCCTCATATGACACTGCGGAAAATG |
| Atu0900-sfGFP Reverse (BamHI) | - | CAGTACGGATCCATTCCCCGGTGG |

## **Table S3: Identified glycans**

| **Schematic** | **Composition** | **Ion [M+H]^+^** | | **Difference (mDa)** |
| --- | --- | --- | --- | --- |
|  |  | **Observed** | **Expected** |  |
|  | GlcNAc-MurNAc | 499.2190 | 499.2133 | 5.7 |
|  | GlcNAc-anhMurNAc | 479.1877 | 479.1872 | 0.5 |
|  | GlcNAc-anhMur | 437.1779 | 437.1765 | 1.4 |
|  | (GlcNAc-MurNAc)_2_ | 977.3907 | 977.3932 | 2.5 |
|  | GlcNAc-MurNAc-GlcNAc-anhMurNAc | 957.3681 | 957.3670 | 1.1 |
|  | GlcNAc-MurNAc-GlcNAc-anhMurN | 915.3548 | 915.3565 | 1.7 |
|  | (GlcNAc-MurNAc)_3_ | 1455.5739 | 1455.5731 | 0.8 |
|  | (GlcNAc-MurNAc)_2_-GlcNAc-anhMurNAc | 1435.5402 | 1435.5469 | 6.7 |
|  | (GlcNAc-MurNAc)_2_-GlcNAc-anhMurN | 1393.5342 | 1393.5363 | 2.1 |
|  | (GlcNAc-MurNAc)_4_ | 1933.7477 | 1933.7530 | 5.3 |
|  | (GlcNAc-MurNAc)_5_ | 804.6530* | 804.6491* | 3.9 |

* [M+3H]^3+^ ion detected
