# Supplementary figures and images for: A peptidoglycan N-deacetylase specific for anhydroMurNAc chain termini in Agrobacterium tumefaciens
Source: J Biol Chem. 2023 Dec 28;300(2):105611. doi: 10.1016/j.jbc.2023.105611 (PMC10838918; doi:10.1016/j.jbc.2023.105611)

A

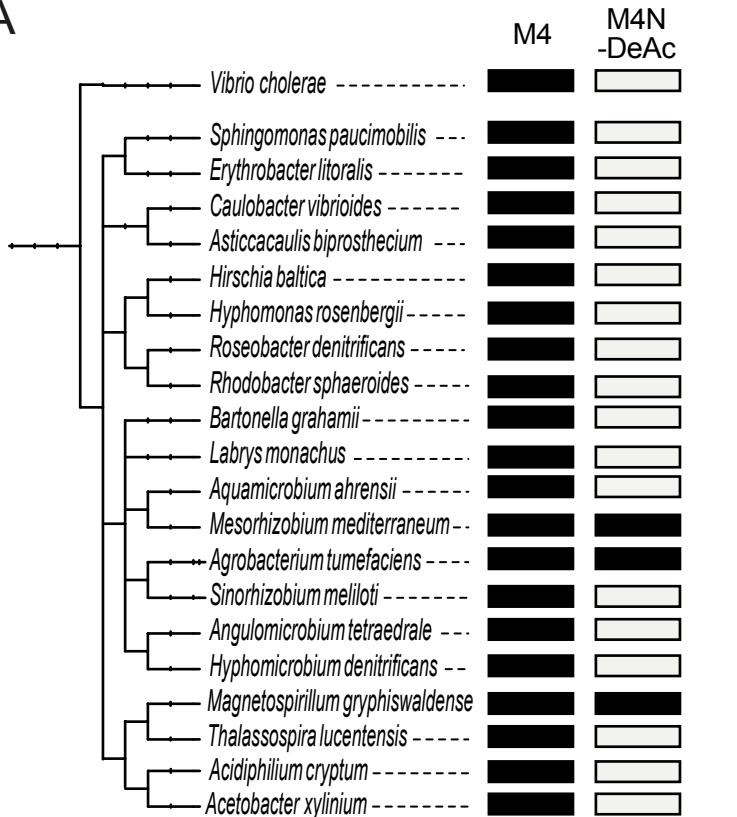

Present  
 Absent

Supplement: Supporting Figure S1 [file mmc5.pdf]

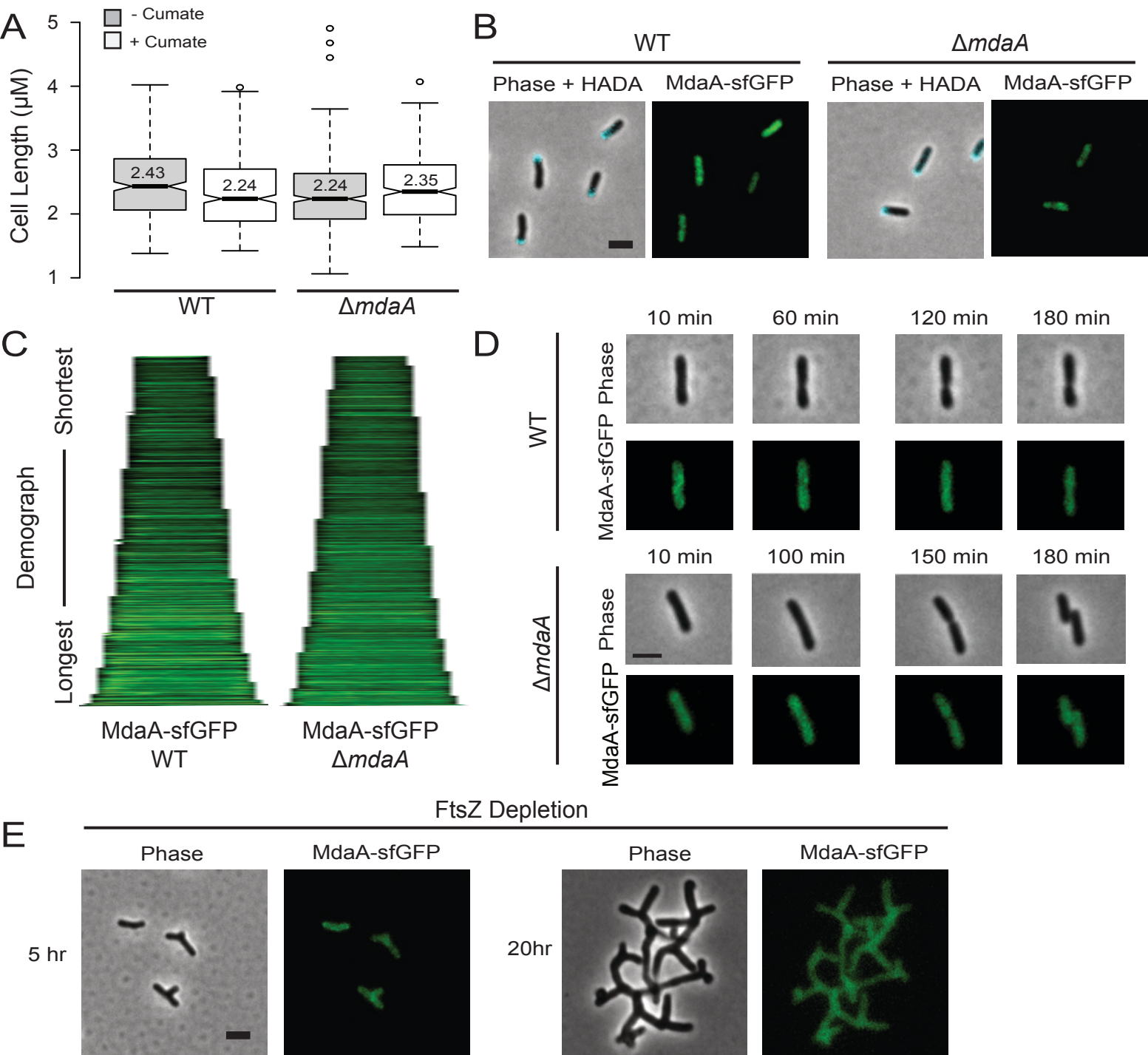

Supplement: Supporting Figure S4 [file mmc8.pdf]
