# Supplementary material for: A peptidoglycan N-deacetylase specific for anhydroMurNAc chain termini in Agrobacterium tumefaciens
Source: J Biol Chem. 2023 Dec 28;300(2):105611. doi: 10.1016/j.jbc.2023.105611 (PMC10838918; doi:10.1016/j.jbc.2023.105611)
Supplement: Supporting Figure S2 [file mmc6.pdf]

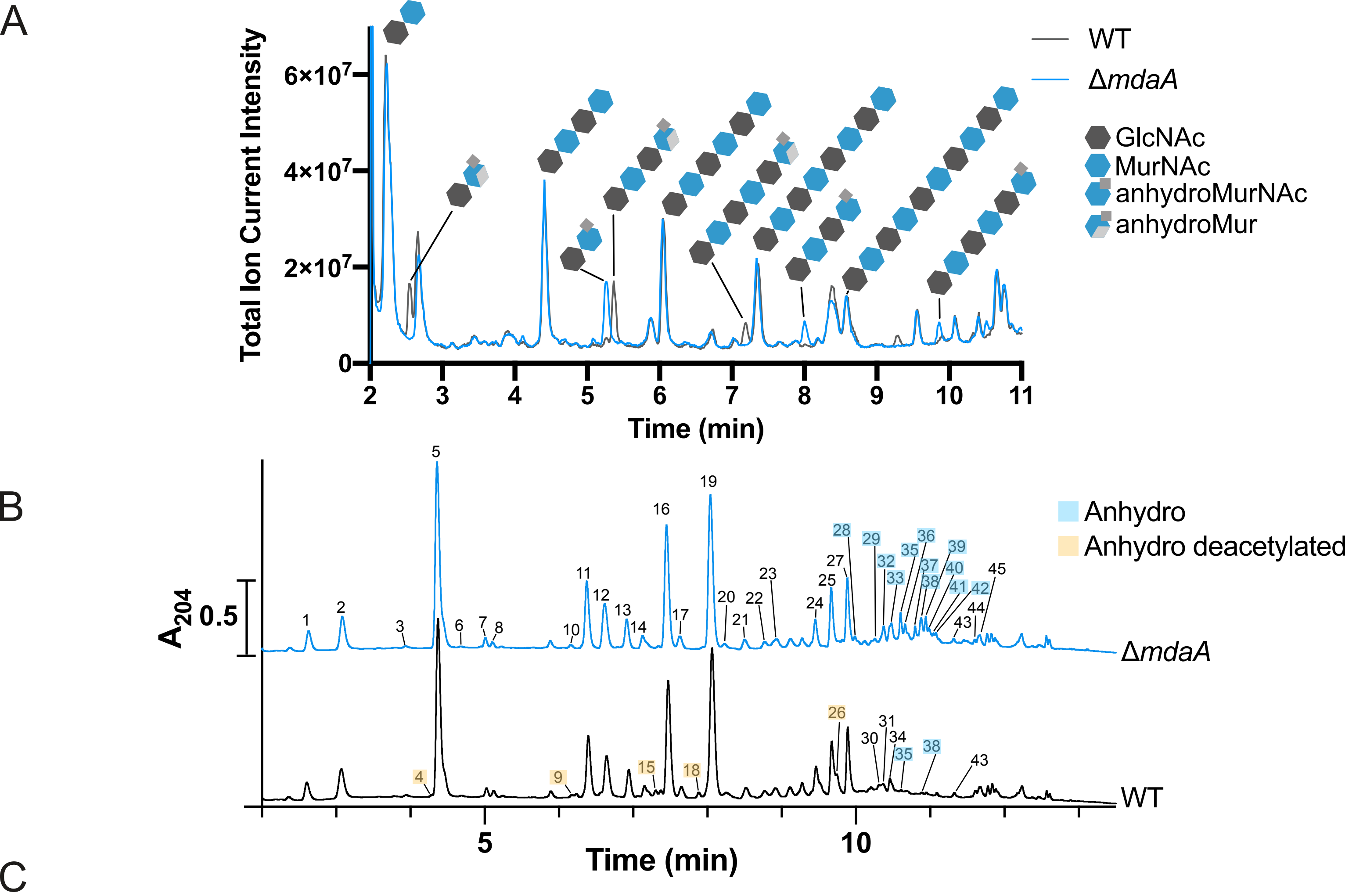

C

Peak	Identity	Proposed Structure	WT	Δ*mdaA*
1	M3	GlcNAc-MurNAc-L-Ala-D-Glu-m-DAP	3.41 ± 0.35	3.16 ± 0.07
2	M4^{G}	GlcNAc-MurNAc-L-Ala-D-Glu-m-DAP-Gly	6.97 ± 0.45	6.52 ± 0.15
3	M5^{G}	GlcNAc-MurNAc-L-Ala-D-Glu-m-DAP-D-Ala-Gly	0.53 ± 0.08	0.45 ± 0.11
4	M4N-DeAc	GlcNAc-anhydroMur-L-Ala-D-Glu-m-DAP-D-Ala	0.14 ± 0.09	0.00 ± 0
5	M4	GlcNAc-MurNAc-L-Ala-D-Glu-m-DAP-D-Ala	32.70 ± 2.19	31.36 ± 1.53
6	M2	GlcNAc-MurNAc-L-Ala-D-Glu	0.30 ± 0.09	0.30 ± 0.25
7	M4	GlcNAc-MurNAc-L-Ala-D-Glu-m-DAP-D-Ala	1.26 ± 0.26	0.92 ± 0.04
8	M5	GlcNAc-MurNAc-L-Ala-D-Glu-m-DAP-D-Ala-D-Ala	0.75 ± 0.18	0.69 ± 0.18
10	D34^{G}N-DeAc	M3-M4^{G}N-DeAc (DAP-DAP crosslink)	0.21 ± 0.01	0.00 ± 0*
11	D34^{G}	M3-M4^{G} (DAP-DAP crosslink)	5.28 ± 0.08	5.13 ± 0.15
12	D33	M3-M3 (DAP-DAP crosslink)	4.08 ± 0.08	3.87 ± 0.07*
13	D44^{G}	M4-M4^{G} (DAP-D-Ala crosslink)	2.26 ± 0.26	1.98 ± 0.16
14	D43	M4-M3 (DAP-D-Ala crosslink)	0.91 ± 0.04	1.06 ± 0.03*
15	D34N-DeAc	M3-M4N-DeAc (DAP-DAP crosslink)	0.22 ± 0.03	0.00 ± 0*
16	D34	M3-M4 (DAP-DAP crosslink)	9.45 ± 0.44	9.33 ± 0.22
17	D34	M3-M4 (DAP-DAP crosslink)	0.84 ± 0.06	0.76 ± 0.01
18	D34N-DeAc	M3-M4N-DeAc (DAP-DAP crosslink)	0.32 ± 0.06	0.00 ± 0*
19	D44	M4-M4 (DAP-D-Ala crosslink)	15.63 ± 1.87	13.81 ± 0.8
20	D44	M4-M4 (DAP-D-Ala crosslink)	0.82 ± 0.15	0.61 ± 0.07
21	T334^{G}	M3-M3-M4^{G} (DAP-DAP crosslinks)	0.71 ± 0.04	0.56 ± 0.06*
22	T333	M3-M3-M3 (DAP-DAP crosslinks)	0.44 ± 0.01	0.38 ± 0.04
23	T335^{G}	M3-M3-M5^{G} (DAP-DAP crosslinks)	0.67 ± 0.03	0.66 ± 0.04
24	T334	M3-M3-M4 (DAP-DAP crosslinks)	1.84 ± 0.18	1.26 ± 0.07*
25	T344	M3-M4-M4 (1×DAP-DAP, 1×DAP-D-Ala crosslinks)	2.43 ± 0.11	2.20 ± 0.1
26	T444N-DeAc	M4-M4-M4N-DeAc (DAP-D-Ala crosslinks)	0.72 ± 0.07	0.00 ± 0*
27	T444	M4-M4-M4 (DAP-D-Ala crosslinks)	2.88 ± 0.39	2.59 ± 0.2
28	D33N	M3-M3N (DAP-DAP crosslink)	0.00 ± 0	0.33 ± 0.05*
29	D34N	M3-M4N (DAP-DAP crosslink)	0.00 ± 0	0.46 ± 0.07*
30	Tt4443	M4-M4-M4-M3 (DAP-D-Ala crosslinks)	0.24 ± 0.07	0.00 ± 0*
31	Tt4443	M4-M4-M4-M3 (DAP-D-Ala crosslinks)	0.34 ± 0.12	0.00 ± 0*
32	D43N	M4-M3N (DAP-D-Ala crosslink)	0.00 ± 0	1.12 ± 0.14*
33	D34N	M3-M4N (DAP-DAP crosslink)	0.00 ± 0	1.65 ± 0.23*
34	Tt4444	M4-M4-M4-M4 (DAP-D-Ala crosslinks)	0.53 ± 0.22	0.00 ± 0*
35	D44N	M4-M4N (DAP-D-Ala crosslink)	0.20 ± 0.09	1.35 ± 0.27*
36	D44N	M4-M4N (DAP-D-Ala crosslink)	0.00 ± 0	1.13 ± 0.41*
37	T334N	M3-M3-M4N (DAP-DAP crosslinks)	0.00 ± 0	0.52 ± 0.26*
38	T344N	M3-M4-M4N (1×DAP-DAP, 1×DAP-D-Ala crosslinks)	0.08 ± 0.05	0.90 ± 0.17*
39	T444N	M4-M4-M4N (DAP-D-Ala crosslinks)	0.08 ± 0.06	0.75 ± 0.11*
40	T444N	M4-M4-M4N (DAP-D-Ala crosslinks)	0.00 ± 0	0.51 ± 0.12*
41	T444N	M4-M4-M4N (DAP-D-Ala crosslinks)	0.00 ± 0	0.24 ± 0.07*
42	T444N	M4-M4-M4N (DAP-D-Ala crosslinks)	0.00 ± 0	0.46 ± 0.22*
43	M4F	GlcNAc-MurNAc-L-Ala-D-Glu-m-DAP-D-Phe	0.52 ± 0.05	0.86 ± 0.79
44	M4F	GlcNAc-MurNAc-L-Ala-D-Glu-m-DAP-D-Phe	0.58 ± 0.02	0.46 ± 0.3
45	M4L	GlcNAc-MurNAc-L-Ala-D-Glu-m-DAP-D-Leu	1.45 ± 0.07	1.46 ± 0.55
