# Supplementary material for: A peptidoglycan N-deacetylase specific for anhydroMurNAc chain termini in Agrobacterium tumefaciens
Source: J Biol Chem. 2023 Dec 28;300(2):105611. doi: 10.1016/j.jbc.2023.105611 (PMC10838918; doi:10.1016/j.jbc.2023.105611)

A

Antibiotic	MIC (μg/mL) WT	MIC (μg/mL) Δ*mdaA*
Ampicillin	32	32
Aztreonam	128	128
Mecillinam	16	16
Imipenem	0.19	0.19
Colistin	0.5	0.5
Cloramphenicol	96	96

B

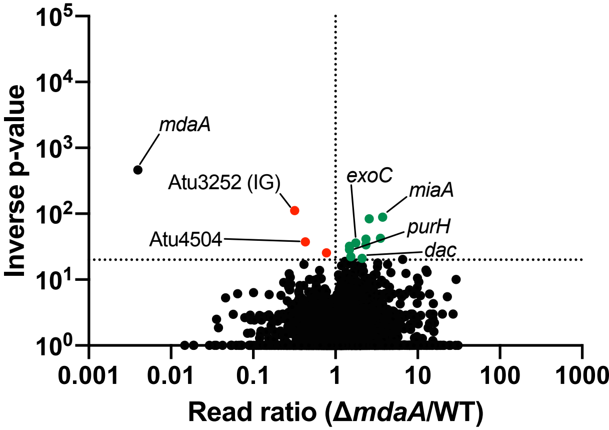

C

Locus	Gene name	Read ratio	p-value	Annotation
IG_Atu3252	-	0.32	0.009	
Atu4504	-	0.43	0.027	Transcriptional regulator, MarR family
IG_Atu3942	-	0.78	0.039	
Atu2823	*purH*	1.48	0.035	Bifunctional purine biosynthesis protein PurH
Atu6127	*traA*	1.49	0.031	Conjugal transfer protein TraA
Atu5136	*blcR*	1.54	0.045	Transcriptional repressor of the blcABC operon
Atu4074	*exoC*	1.77	0.028	Phosphoglucomutase
Atu1505	*dac*	2.12	0.048	Penicillin-binding protein
Atu1056	-	2.35	0.024	TIGR02301 family protein
IG_Atu2172	-	2.36	0.030	
Atu0439	-	2.58	0.012	Uncharacterized protein
Atu0789	-	3.53	0.024	Uncharacterized protein
Atu2039	*miaA*	3.76	0.011	tRNA dimethylallyltransferase
Atu1835	-	6.60	0.049	Uncharacterized protein

D

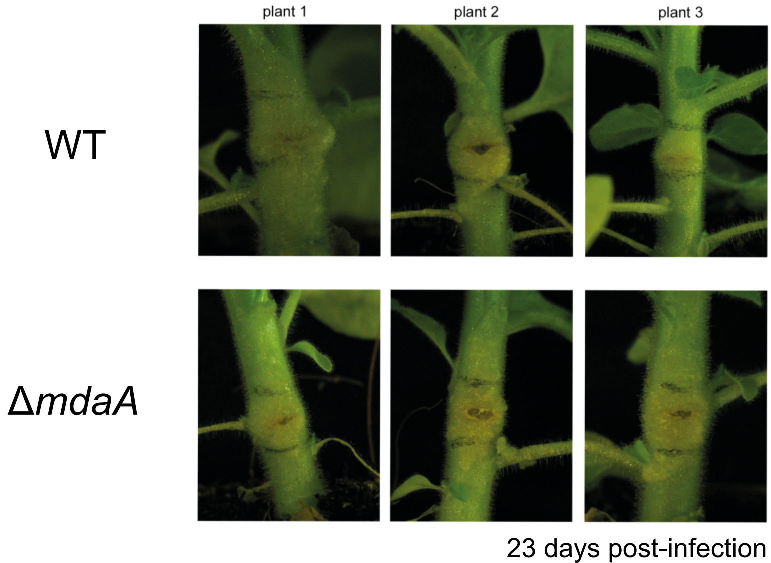

Supplement: Supporting Figure S3 [file mmc7.pdf]
